# Thiophene/selenophene-based S-shaped double helicenes: regioselective synthesis and structures

**DOI:** 10.3762/bjoc.18.81

**Published:** 2022-07-08

**Authors:** Mengjie Wang, Lanping Dang, Wan Xu, Zhiying Ma, Liuliu Shao, Guangxia Wang, Chunli Li, Hua Wang

**Affiliations:** 1 Engineering Research Center for Nanomaterials, Henan University, Kaifeng, 475004, China

**Keywords:** crystal structure, double helicene, regioselective synthesis, selenophene, thiophene

## Abstract

2,5-Di(trimethylsilanyl)dithieno[2,3-*b*:3′,2′-*d*]thiophene ((TMS)_2_-*bb*-**DTT**), 2,5-di(trimethylsilanyl)diseleno[2,3-*b*:3′,2′-*d*]thiophene ((TMS)_2_-*bb*-**DST**), and 2,5-di(trimethylsilanyl)diseleno[2,3-*b*:3′,2′-*d*] selenophene ((TMS)_2_-*bb*-**DSS**) were used as starting materials to synthesize three S-shaped double helicenes (i.e., **DH**-**1**, **DH**-**2**, and **DH**-**3**) through monobromination, formylation, the Wittig reaction, and double oxidative photocyclization. The photocyclization was a highly regioselective process. The molecular structures of **DH**-**1** and **DH**-**2** were confirmed by X-ray single-crystal analysis. Multiple intermolecular interactions, such as C–S, C–Se, S–S, S–Se, and Se–Se, were observed in the crystal packing structures of these compounds. Spectroscopic results and our previous work showed that the combination of molecular structure change and heteroatom replacement from S to Se could precisely modulate molecular energy levels.

## Introduction

Given their esthetically pleasing helical structures, inherent helical chirality, and extended π-conjugation, helicenes have attracted extensive research attention. Helicenes are generally divided into carbohelicenes and heterohelicenes. The rapid development of carbohelicenes has led to the synthesis of double, triple, quadruple, quintuple, and sextuple molecules, the chiro-optical properties of these molecules, such as their circular dichroism (CD) and circularly polarized luminescence (CPL), have also been widely studied [1–8]. The development of thiahelicene, a class of typical heterohelicenes, has led to the preparation of symmetric thiophene-based [5]-, [7]-, [9]-, and [11]helicenes [9–16], unsymmetric thiophene-based [7]helicenes [17], and thiophene-based double helicenes with spiro-silicon atoms [18], “saddle” formed 8π annulene [12], and twisted naphthalene as central spacers [19].

As its close analogue, selenophene has properties very similar to those of thiophene. Fused aromatic compounds containing selenophene units show favorable optical and electrochemical properties and improved charge transport characteristics in the solid state mainly because such fused aromatic compounds often undergo increased Se–Se interactions, which confer ordering at the molecular scale and, thus, lead to well-aligned solid-state packing and excellent charge-transport properties [20–21].

However, as an important type of heteroacenes, fused selenophenes have rarely been reported in the literature because their synthesis is extremely challenging. The first seven-ring-fused heteroacene containing selenophene was synthesized through the intramolecular triple cyclization of bis(*o*-haloaryl)diacetylene by Yamaguchi in 2005 [22]. Using a similar method, Takimiya reported the synthesis of six-ring-fused and four-ring-fused heteroacenes containing selenopheno[3,2-*b*]selenophene in 2007 and 2009, respectively [23–24]. Five years later, Cheng reported the synthesis of two types of five-ring-fused isomers of diselenopheno[2,3-*b*:7,6-*b*′]fluorene and diselenopheno[3,2-*b*:6,7-*b*′]fluorene through the cyclization of terminal acetylene as well as six types of biselenophene-based fused tricyclic derivatives [25–26]. In 2017, we reported the first member of diselenoselenophenes (**DSS**s), 2,5-di(trimethylsilanyl)diseleno[2,3-*b*:3′,2′-*d*]selenophene ((TMS)_2_-*bb*-**DSS**), from which the TMS group could easily be removed by trifluoroacetic acid and replaced by bromine [27]. Another isomer of **DSS**, diseleno[3,2-*b*:2′,3′-*d*]selenophene (*tt*-**DSS**) has been successively synthesized from selenophene [28–29].

**Figure 1:**
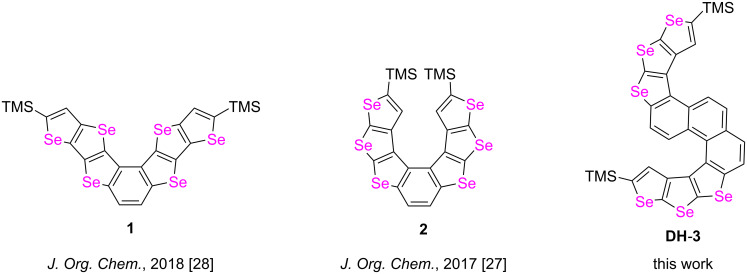
Molecular structures of bull horn-shaped heteroacene **1**, selenophene-based [7]helicene **2** and novel compound **DH**-**3**.

Among the limited fused aromatic compounds containing selenophene currently available, helicenes have received relatively little attention [27]. In our previous work, bull horn-shaped selenophene-based heteroacenes (**1**, Figure 1) and selenophene-based [7]helicene (**2**, Figure 1) were synthesized from the (TMS)_2_-substituted selenophene triacenes, 2,5-di(trimethylsilanyl)diseleno[3,2-*b*:2′,3′-*d*]selenophene ((TMS)_2_-*tt*-**DSS**) and (TMS)_2_-*bb*-**DSS**, respectively. In this work, the novel S-shaped double helicene **DH**-**3** (Figure 1), which is based on selenophene units, was constructed as a new member of the selenohelicene family (Figure 1).

In this synthetic study, regioselective double oxidative photocyclization was observed during the construction of three S-shaped double helicenes **DH**-**1**–**3** based on thiophene/selenophene. From **DH**-**1** to **DH**-**3**, sulfur atoms in the molecular framework were gradually replaced by selenium atoms. The crystal structures of **DH**-**1** and **DH**-**2** and spectroscopic features of **DH**-**1**–**3** were then studied. Finally, the reaction sites of oxidative photocyclization, energy levels, and the electron cloud distribution of the highest occupied molecular orbitals (HOMOs) and the lowest unoccupied molecular orbitals (LUMOs) are predicted.

## Results and Discussion

### Synthesis of **5a–c** and **DH-1–3**

**Scheme 1:**
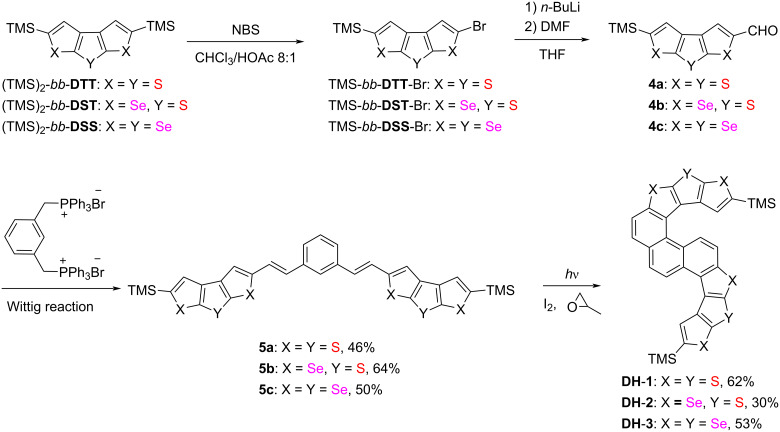
Synthetic route to S-shaped double helicenes **DH**-**1**–**3**.

The synthetic route of 1,3-bis(2-(5-(trimethylsilyl)dithieno-[2,3-*b*:3',2'-*d*]thiophen-2-yl)vinyl)benzene (**5a**), 1,3-bis(2-(5-(trimethylsilyl)diselenopheno[2,3-*b*:3',2'-*d*]thiophen-2-yl)vinyl)benzene (**5b**), 1,3-bis(2-(5-(trimethylsilyl)diselenopheno[2,3-*b*:3',2'-*d*]selenophen-2-yl)vinyl)benzene (**5c**), and S-shaped double helicenes **DH-1**–**3** is shown in Scheme 1. The double oxidative photocyclization of **5a**–**c** is the key step in the synthesis of **DH**-**1**–**3** because oxidative photocyclization induces double radicals on a double bond, which led to the C=C bond rotation along the resulting single C(radical)–C(radical) bond and randomly directed annelated products [30]. Moreover, compounds **5a**–**c** bear two C=C bonds, which may lead to more complex photocyclization products.

5-(Trimethylsilyl)dithieno[2,3-*b*:3′,2′-*d*]thiophene-2-carbaldehyde (**4a**) [19], 5-(trimethylsilyl)diseleno[2,3-*b*:3′,2′-*d*]thiophene-2-carbaldehyde (**4b**) [27], and 5-(trimethylsilyl)diseleno[2,3-*b*:3′,2′-*d*]selenophene-2-carbaldehyde (**4c**) [27] were prepared via monobromination and formylation reactions with (TMS)_2_-*bb*-**DTT**, (TMS)_2_-*bb*-**DST**, and (TMS)_2_-*bb*-**DSS** as starting materials according to the literature. In (TMS)_2_-*bb*-**DTT**, (TMS)_2_-*bb*-**DST**, and (TMS)_2_-*bb*-**DSS**, the notation *bb* means that the positions of the heteroatoms of the outer two heterocycles are on the same side as those of the middle heterocycle. Taking dithienothiophene as example, according to the positions of sulfur atoms, there are six isomers of dithienothiophene, in which the sulfur atoms on the same side is defined as *bb* by us. The double Wittig reaction of **4a** and **4b** with 1,3-phenyldimethyltriphenylphosphonium bromide afforded compounds **5a** and **5b** with yields of 46% and 64%, respectively. After the double Wittig reaction of **4c** we obtained a mixture of *cis* and *trans* isomers of **5c** with the total yield of 50%. According to the results of ^1^H NMR the ratio of *cis* and *trans* isomers was approximately 1:0.25 (Scheme 1).

**Figure 2:**
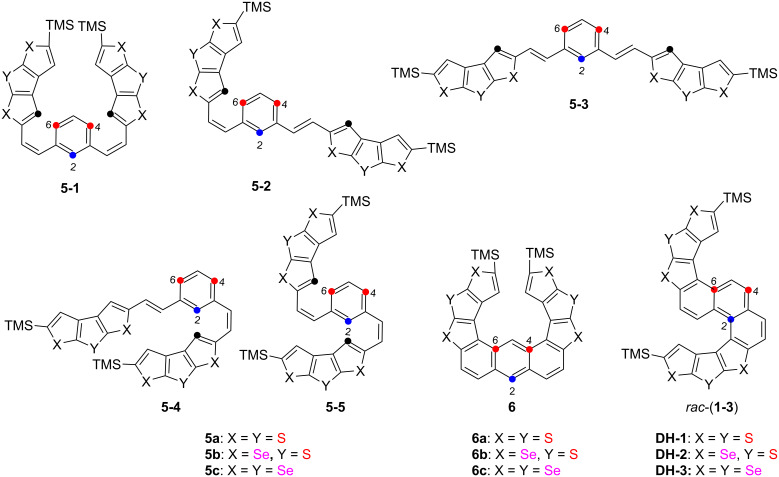
Five kinds of isomer structures of **5** and two kinds of possible oxidative photocyclization product structures of **6** and **DH**-**1**–**3**.

Compounds **5** had five isomers and three reaction sites (2, 4, and 6-positions in the benzene moiety) during oxidative photocyclization. Irradiation of **5a**–**c** resulted in oxidative photocyclization products with two types of configurations wherein two benzene rings were closed in the same and opposite direction, such as **DH**-**1**–**3** and **6** (Figure 2). However, after the double oxidative photocyclizations of **5a**–**c** in the presence of iodine and propylene oxide in dry toluene through irradiation by a 450 W Hg medium-pressure lamp for 1.5 h, only one type of ring-closing product with two benzene rings formed in the same direction, i.e., S-shaped double helicenes **DH**-**1**–**3** were obtained in yields of 62%, 30%, and 53%, respectively (Figure 2).

The double oxidative photocyclization reaction sites of **5** were predicted by the orbital-weighted Fukui function in Multiwfn using Gaussian 09 [31–32] at the B3LYP/6-31G** [33] level of theory to verify the reaction-site selectivity of oxidative photocyclization further. Results showed that the conformations of **5a** are varied, but the orbital-weighted Fukui function is not connected to conformation. In the orbital-weighted Fukui function, the larger the isosurface distribution, the higher the activity of reactions. Thus the 4 and 6-positions of benzene are the most likely sites for reaction (see Supporting Information File 1, Figure S21). However, after the formation of the naphthalene ring, the α-position is the most likely site for reaction (Supporting Information File 1, Figure S22). Therefore, after the double oxidative photocyclization of compound **5a**, product **DH**-**1** is mainly obtained. The predicted result of the double oxidative photocyclizations of **5a** is consistent with the experimental result, that is, an S-shaped double helicene can be selectively obtained through the double oxidative photocyclization of compound **5a**.

### Crystallographic analyses of **DH-1** and **DH-2**

**Figure 3:**
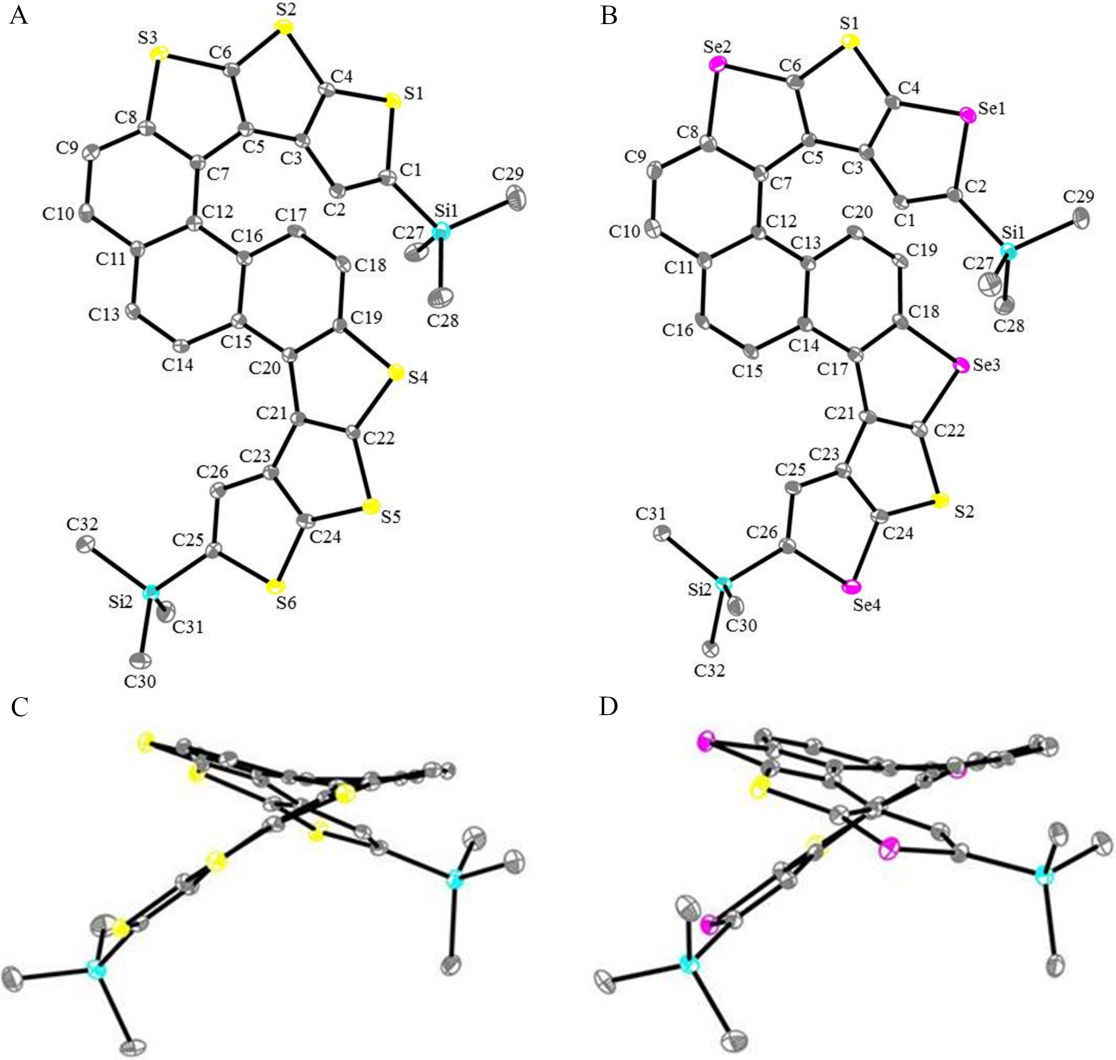
Molecular structures and side view for **DH**-**1** and **DH**-**2**. A and B are molecular structures for **DH**-**1** and **DH**-**2**. C and D are side views for **DH**-**1** and **DH**-**2**. Carbon, sulfur, selenium, and silicon atoms are depicted with thermal ellipsoids set at the 30% probability level and all hydrogen atoms are omitted for clarity.

The molecular structures of **DH**-**1** and **DH**-**2** were confirmed by single-crystal analysis (Figure 3). Both **DH**-**1** and **DH**-**2** belong to the triclinic space group *P*-1. After double oxidative photocyclizations of **5a** and **5b**, **DH**-**1** and **DH**-**2** are compressed into S-shaped double helical structures (Figure 3A and B), which consist of one [5]helicene and one [6]helicene. The two helicenes have the same configuration and bend toward the same direction on the same side of the shared naphthalene ring (Figure 3C and D). Both products **DH**-**1** or **DH**-**2** feature a pair of enantiomers *MM* and *PP* in their unit cell (see Supporting Information File 2, Figures S2 and S6). The crystal parameters of **DH**-**1** and **DH**-**2** are shown in Table 1. The replacement of sulfur with selenium in **DH**-**1** and **DH**-**2** leads to turn angles in-plane and helix climbs of [5]helicene and [6]helicene of **DH**-**1** and **DH**-**2** significantly change (Table 1, Figure 3).

**Table 1:** Crystal parameters of helicenes **DH**-**1** and **DH**-**2**.

Compound		Dihedral angle (^o^)^a^	Torsion angle (^o^)^b^	Turn angle in-plane (^o^)^c^	Helix climbs (Å)^d^

**DH**-**1**	[5]helicene	25.00	38.64	231.1	0.932
	[6]helicene	50.61	64.38	273.6	2.424
**DH**-**2**	[5]helicene	25.01	37.27	230.9	0.879
	[6]helicene	48.16	64.12	279.3	2.462

^a^Dihedral angle between two terminal rings of helicene. ^b^The sum of the three ([5]helicene) or four ([6]helicene) distortion angles. ^c^The angle of turns in-plane. ^d^Internal helix climb height.

Each of the two **DH**-**1** molecules (blue and red molecules in Figure S3 of Supporting Information File 2) in the unit cell interacts with six adjacent molecules. For example, in Figure S3 (Supporting Information File 2), the blue molecule interacts with six adjacent molecules through multiple interactions, such as, S···S (3.44 Å), S···H (2.94 Å, 2.91 Å, 2.97 Å), H···H (2.31 Å), C···C (3.31 Å), S···C (3.45 Å), and C···H (2.90 Å). Each of the two **DH**-**2** molecules (blue and red molecules, Supporting Information File 2, Figure S7) in the unit cell interact with eight adjacent molecules through multiple interactions, such as, C···C (3.40 Å), Se···H (3.09 Å), H···H (2.26 Å), C···H (2.90 Å), Se···Se (3.62 Å), and Se···S (3.58 Å). However, in contrast to **DH**-**1**, **DH**-**2** exhibits multiple S···H (2.88 Å), S···C (3.45 Å), and Se···H (2.94 Å) interactions between two molecules in the unit cell. These multiple interactions confer **DH**-**1** and **DH**-**2** with a regular arrangement featuring multi channels (Figures S4 and S8 in Supporting Information File 2), which is a suitable characteristic for helicene compounds used as supramolecular self-assembly units [34–41].

### Spectroscopic features of **DH-1–3**

**Figure 4:**
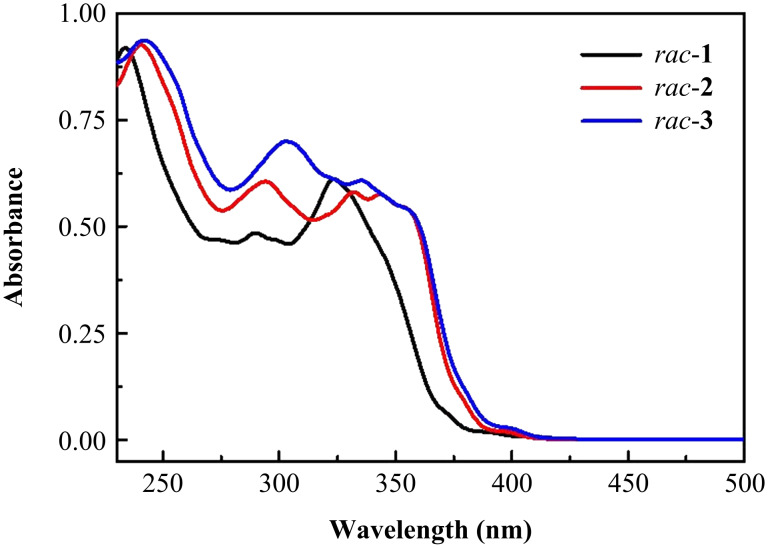
UV–vis absorption spectra of **DH**-**1**–**3** in CH_2_Cl_2_ ([*c*] = 1 × 10^−5^ M).

The UV–vis absorption spectra of **DH**-**1**–**3** in dichloromethane are shown in Figure 4. In general, the UV–vis absorption spectra of **DH**-**1**–**3** are generally similar in shape and exhibit three major absorption bands within 230–280 nm (band-I), 280–330 nm (band-II), and 304–414 nm (band-III) (Figure 4). Progressive red-shifts in the absorption spectra of band-I, band-II, and band-III occur with increasing number of selenium atoms. In band-I, compounds **DH**-**1**–**3** show a maximum absorption peak at 232, 240, and 242 nm, respectively. In band-II and band-III, helical distortion and possible conjugation through heteroatoms (e.g., sulfur and selenium atoms) in **DH**-**1**–**3** may increase π-electron delocalization, leading to a red-shifted broad absorption. The maximum absorption peaks of **DH**-**1**–**3** appear at 268, 275, and 279 nm, respectively, in band-II and at 323, 331, and 336 nm, respectively, in band-III. Thus, the optical band gaps estimated from the absorption edges gradually decrease from **DH**-**1** to **DH**-**2** to **DH**-**3**, and are equal to 3.08, 3.01, and 2.98 eV, respectively. This change trend is consistent with the calculated results, which are 3.97, 3.83, and 3.81 eV for **DH**-**1**, **DH**-**2**, and **DH**-**3**, respectively (see Table S2 in Supporting Information File 1). However, the optical band gaps of **1**, **2**, and **DH**-**3** obviously differ because of changes in their molecular configuration and equal to 2.86, 3.15, and 3.81 eV, respectively [27–28]. As the number of selenium atoms increases from **DH**-**1** to **DH**-**3**, the fluorescence intensity (Figure S19 in Supporting Information File 1) and the fluorescence quantum yield (Φ_F_, Figure S20, Table S1 in Supporting Information File 1) of the molecules also decrease (Figure 4).

## Conclusion

In summary, the key step of regioselective double oxidative photocyclization was successfully employed in the preparation of three S-shaped double helicenes, namely, **DH**-**1**, **DH**-**2** and **DH**-**3** with (TMS)_2_-*bb*-**DTT**, (TMS)_2_-*bb*-**DST**, and (TMS)_2_-*bb*-**DSS** as starting materials. The synthetic method described in this research not only provides a method for the synthesis of S- shaped double helicenes but also enriches the family of selenophene helicenes. Multiple intermolecular interactions and regular arrangement in the crystal packing structures of **DH**-**1** and **DH**-**2** indicate that these compounds may be used as supramolecular self-assembly units. Changes in molecular structure may substantially modulate the optical band gap of heteroacenes, and the replacement of heteroatoms from S to Se could fine-tune their optical band gap [18,27–28]. Thus, the combination of molecular structure modification and atom replacement could be a viable strategy, for the precise modulation molecular energy levels and yield molecules with strong application potential in organic functional materials, such as OFETs, and CPLs, among others.

## Experimental

### General procedures and materials

Tetrahydrofuran (THF) for use on vacuum line was freshly distilled from sodium/benzophenone prior to use. *n*-BuLi (hexane) were obtained from Energy Chemical; prior to use, its concentration was determined by titration with *N*-pivaloyl-*o*-toluidine [42]. Column chromatography was carried out on silica gel (300–400 mesh). Analytical thin-layer chromatography was performed on glass plates of silica gel GF-254 with detection by UV. Standard techniques for synthesis under inert atmosphere and Schlenk glassware equipped with an 8 mm PTFE vacuum stopcock, were employed. All starting materials and reagents were commercially available.

^1^H NMR and ^13^C{^1^H} NMR spectra were recorded on 300 or 400 MHz NMR instruments using CDCl_3_ as solvent. The chemical shift references were as follows: (^1^H) CDCl_3_, 7.26 ppm (CHCl_3_); (^13^C{^1^H}) CDCl_3_, 77.00 ppm (CDCl_3_). IR spectra were obtained using an FTIR instrument. MS analysis was carried out on mass spectrometers equipped with EI (70 eV). HRMS analysis was carried out on a mass spectrometer equipped with DART-FT-ICR and MALDI-TOF-CHCA. Melting-point determination was taken on a Melt-Temp apparatus and mp are uncorrected. The X-ray crystallographic analyses were performed using crystals of compounds **DH**-**1** and **DH**-**2** with sizes of 0.14 × 0.12 × 0.08, 0.21 × 0.17 × 0.12 mm^3^, respectively. The intensity data were collected with the ω scan mode (296 K) on a diffractometer with a CCD detector using Cu Kα radiation (λ = 1.54184 Å). The data were corrected for Lorentz and polarization effects, and absorption corrections were performed using the SADABS program [43]. The crystal structures were solved using the SHELXTL program and refined using full-matrix least-squares [44]. Further details are in the deposited CIF files. Slow evaporation of solutions of **DH**-**1** and **DH**-**2** in CHCl_3_/CH_3_OH 5:1 (v/v) were employed for growing single crystals. The fluorescence quantum yields (Φ_F_) of **DH**-**1**–**3** are characterized in dichloromethane with quinine sulfate in 0.1 N H_2_SO_4_ as the control.

### Synthesis of **5a–c** and **DH-1–3**

#### Synthesis of 1,3-bis(2-(5-(trimethylsilyl)dithieno[2,3-*b*:3',2'-*d*]thiophen-2-yl)vinyl)benzene (**5a**)

*n*-BuLi (0.06 mL, 2.50 M in hexane, 0.168 mmol, 2.5 equiv) was added dropwise to 1,3-bis[methyl(bromotriphenylphosphonium)] (53.3 mg, 0.067 mmol) in THF (40 mL) at −78 °C. After 2 h at −78 °C, a solution of **4a** (41.0 mg, 0.138 mmol, 2.05 equiv) in THF (5 mL) was added at −78 °C, the mixture kept for 1 h, and then the reaction mixture was warmed up slowly to ambient temperature overnight. The reaction mixture was quenched with CH_3_OH and extracted with CH_2_Cl_2_ (3 × 10 mL). The organic layer was washed with saturated NaCl (20 mL) and water (2 × 20 mL), and then dried over MgSO_4_. The residue was purified by column chromatography (eluent: hexane/CH_2_Cl_2_ 3:1 (v/v) and recrystallized from CHCl_3_/CH_3_OH to yield **5a** (20.9 mg, 46%) as a yellow solid; mp > 300 °C; ^1^H NMR (400 MHz, CDCl_3_) δ (ppm) 7.58 (s, 1H), 7.45 (s, 2H), 7.41–7.36 (m, 3H), 7.34 (s, 2H), 7.31 (d, *J* = 16.0 Hz, 2H), 6.93 (d, *J* = 16.0 Hz, 2H), 0.39 (s, 18H); ^13^C{^1^H} NMR (100 MHz, CDCl_3_) δ (ppm) 145.3, 144.7, 143.7, 140.7, 138.4, 137.8, 137.2, 129.1, 127.8, 125.5, 124.9, 124.4, 122.8, 118.0, −0.1; IR (KBr): 3018, 2958, 2849, 1631, 1408, 1360, 945, 837 cm^−1^; EIMS (70 eV) *m*/*z*: [M]^+^ 662.18 (40); HRMS-MALDI (*m*/*z*): [M]^+^ calcd for C_32_H_30_S_6_Si_2_, 662.0216; found, 662.0205.

1,3-Bis(2-(5-(trimethylsilyl)diselenopheno[2,3-*b*:3',2'-*d*]thiophen-2-yl)vinyl)benzene (**5b**) was synthesized according to the procedure described for compound **5a**.

**5b**: yellow solid in yield of 64% (46.2 mg); mp > 300 °C; ^1^H NMR (300 MHz, CDCl_3_) δ (ppm) 7.73 (s, 2H), 7.55 (d, *J* = 12.9 Hz, 3H), 7.38–7.32 (m, 5 H), 6.79 (d, *J* = 15.6 Hz, 2H), 0.38 (s, 18H); ^13^C{^1^H} NMR (100 MHz, CDCl_3_) δ (ppm) 151.6, 149.7, 144.5, 142.6, 141.1, 137.01, 136.98, 129.2, 128.6, 127.5, 125.6, 124.9, 124.4, 121.0, 0.2; IR (KBr): 3011, 2949, 2891, 1627, 1427, 1367, 920, 831 cm^−1^; DARTMS (*m*/*z*): [M + H]^+^ 854.8 (75); HRMS-DART (*m*/*z*): [M + H]^+^ calcd for C_32_H_31_S_2_Se_4_Si_2_, 854.8071; found, 854.8067.

1,3-Bis(2-(5-(trimethylsilyl)diselenopheno[2,3-*b*:3',2'-*d*]selenophen-2-yl)vinyl)benzene (**5c**) was synthesized according to the procedure described for compound **5a**.

Mixture of *cis* and *trans* isomers **5c**: yellow solid in yield of 50% (24.4 mg); mp > 300 °C; ^1^H NMR (400 MHz, CDCl_3_) δ (ppm): 7.76 (s, *trans*-), 7.73 (s, *cis*- and *trans*-), 7.72 (s, *cis*- and *trans*-), 7.57 (s, *cis*- and *trans*-), 7.56 (s, *trans*-), 7.49–7.33 (m, *trans*-, *cis*- and *trans*-), 7.30 (d, *J* = 16.0 Hz, *cis*- and *trans*-), 6.90 (d, *J* = 12.0 Hz, *cis*- and *trans*-), 6.79 (d, *J* = 16.0 Hz, *trans*-), 6.78 (d, *J* = 16.0 Hz, *trans*- and *trans*-), 6.65 (d, *J* = 12.0 Hz, *cis*- and *trans*-), 0.38 (s, *trans*-), 0.36 (s, *cis*- and *trans*-), 0.35 (s, *cis*- and *trans*-), the ratio of integral areas of the peaks at 7.76, 7.73, and 7.72 ppm is 1.0:0.25; ^13^C{^1^H} NMR (125 MHz, CDCl_3_) δ (ppm): 152.78, 152.70, 152.62, 151.28, 151.23, 147.31, 146.49, 146.34, 143.49, 143.47, 141.84, 140.87, 140.81, 140.72, 139.02, 137.66, 137.50, 137.43, 135.39, 135.36, 129.74, 129.30, 129.28, 129.23, 129.05, 128.83, 128.42, 128.35, 127.50, 126.97, 126.28, 125.68, 125.20, 125.10, 125.04, 124.85, 124.63, 122.61, 122.51, 0.43, 0.41; IR (KBr): 3010, 2951, 2889, 1624, 1413, 1369, 908, 833 cm^−1^; MALDIMS (*m*/*z*): [M]^+^ 949.8; HRMS-DART-FT (*m*/*z*): [M]^+^ calcd for C_32_H_30_Se_6_Si_2_, 949.6894; found, 949.6887.

#### Synthesis of **DH-1**

To a solution of **5a** (9.6 mg, 0.014 mmol) in dry toluene (6 mL), iodine (7.3 mg, 0.028 mmol, 2.0 equiv) and excess propylene oxide were added. The reaction solution was irradiated with a 450 W unfiltered Hg medium-pressure lamp for 1.5 h. The reaction was quenched with saturated Na_2_SO_3_ solution (5 mL). The reaction mixture was extracted with CH_2_Cl_2_ (3 × 5 mL), washed with H_2_O (3 × 10 mL), and then dried over MgSO_4_. After removing the solvent in vacuum, the crude product was purified by PTLC with petrol ether (60–90 °C) and hexane/CH_2_Cl_2_ 5:1 (v/v) as developer to yield **DH**-**1** (5.9 mg, 62%) as a light-yellow solid; mp > 300 °C; ^1^H NMR (400 MHz, CDCl_3_) δ (ppm) 9.09 (d, *J* = 8.8 Hz, 1H), 8.51 (d, *J* = 8.8 Hz, 1H), 8.22 (s, 1H), 8.11 (d, *J* = 9.2 Hz, 1H), 8.04 (d, *J* = 8.4 Hz, 1H), 7.90 (d, *J* = 8.4 Hz, 1H), 7.78 (d, *J* = 8.8 Hz, 1H), 6.62 (s, 1H), 0.48 (s, 9H), 0.14 (s, 9H); ^13^C{^1^H} NMR (100 MHz, CDCl_3_) δ (ppm) 144.6, 144.1, 143.19, 143.18, 142.1, 141.7, 141.6, 141.4, 141.3, 141.1, 135.0, 133.4, 130.6, 129.8, 129.0, 128.5, 127.53, 127.46, 127.3, 127.0, 126.7, 126.4, 124.3, 123.7, 121.5, 119. 7, 0.0, −0.4; IR (KBr): 3045, 2952, 2860, 1645, 1487, 1410, 972, 837 cm^−1^; EIMS (70 eV) *m*/*z*: [M]^+^ 657.87 (30); HRMS-MALDI (*m*/*z*): [M]^+^ calcd for C_32_H_26_S_6_Si_2_ 657.9897; found, 657.9892.

**DH**-**2** was synthesized according to the procedure described for compound **DH**-**1**.

**DH**-**2**: light-yellow solid in yield of 30% (15.8 mg); mp > 300 °C; ^1^H NMR (400 MHz, CDCl_3_) δ (ppm) 8.92 (d, *J* = 8.8 Hz, 1H), 8.54 (s, 1H), 8.32 (d, *J* = 8.4 Hz, 1H), 8.04 (d, *J* = 8.0 Hz, 1H), 7.95 (d, *J* = 8.8 Hz, 1H), 7.80 (d, *J* = 8.0 Hz, 1H), 7.66 (d, *J* = 8.8 Hz, 1H), 6.66 (s, 1H), 0.47 (s, 9H), −0.01 (s, 9H); ^13^C{^1^H} NMR (100 MHz, CDCl_3_) δ (ppm) 149.9, 146.9, 145.7, 145.1, 144.8, 144.3, 142.7, 142.6, 139.8, 139.4, 139.2, 137.5, 132.8, 130.8, 130.6, 130.2, 129.59, 129.58, 128.4, 128.1, 126.7, 125.6, 124.5, 124.3, 124.2, 122.2, 0.3, −0.2; IR (KBr): 3060, 2949, 2896, 1632, 1489, 1402, 933, 829 cm^−1^; DARTMS (*m*/*z*): [M + H]^+^ 850.8 (45); HRMS-DART (*m*/*z*): [M + H]^+^ calcd for C_32_H_27_S_2_Si_2_Se_4_, 850.7753; found, 850.7743.

**DH**-**3** was synthesized according to the procedure described for **DH**-**1**.

**DH**-**3**: light yellow solid in yield of 53% (19.5 mg); mp 265.1–266.9 °C; ^1^H NMR (300 MHz, CDCl_3_) δ (ppm) 8.85 (d, *J* = 8.7 Hz, 1H), 8.56 (s, 1H), 8.22 (d, *J* = 8.7 Hz, 1H), 8.06 (d, *J* = 8.4 Hz, 1H), 7.92 (d, *J* = 9.0 Hz, 1H), 7.81 (d, *J* = 8.7 Hz, 1H), 7.63 (d, *J* = 8.7 Hz, 1H), 6.68 (s, 1H), 0.45 (s, 9H), −0.11 (s, 9H); ^13^C{^1^H} NMR (150 MHz, CDCl_3_) δ (ppm) 151.0, 148.0, 147.4, 147.0, 145.9, 143.9, 142.6, 141.1, 140.9, 139.34, 139.26, 138.9, 134.1, 132.3, 131.7, 130.7, 130.2, 129.3, 128.8, 128.0, 126.7, 125.0, 124.9, 124.1, 123.9, 121.9, 0.4, −0.3; IR (KBr): 3047, 2947, 2895, 1633, 1504, 1409, 925, 831 cm^−1^; DARTMS (*m*/*z*): [M]^+^ 945.8; HRMS-DART-FT (*m*/*z*): [M]^+^ calcd for C_32_H_26_Se_6_Si_2_, 945.6564; found, 945.6564.

## Supporting Information

File 1 Spectral and computational data.

File 2 Supporting crystallographic information for compounds **DH**-**1** and **DH**-**2**.

File 3 Title CIF file for compound **DH-1**.

File 4 Title CIF file for compound **DH-2**.
