# Supplementary material for: Thiophene/selenophene-based S-shaped double helicenes: regioselective synthesis and structures
Source: Beilstein J Org Chem. 2022 Jul 8;18:809–17. doi: 10.3762/bjoc.18.81 (PMC9273980; doi:10.3762/bjoc.18.81)
Supplement: File 1 — Spectral and computational data. [file Beilstein_J_Org_Chem-18-809-s001.pdf]

BEILSTEIN JOURNAL OF ORGANIC CHEMISTRY

Supporting Information

for

Thiophene/selenophene-based S-shaped double helicenes: regioselective synthesis and structures

Mengjie Wang, Lanping Dang, Wan Xu, Zhiying Ma, Liuliu Shao, Guangxia Wang, Chunli Li and Hua Wang

Beilstein J. Org. Chem. **2022,** *18,* 809–817. doi:10.3762/bjoc.18.81

Spectral and computational data

License and Terms: This is a supporting information file under the terms of the Creative Commons Attribution License (https://creativecommons.org/licenses/by/4.0). Please note that the reuse, redistribution and reproduction in particular requires that the author(s) and source are credited and that individual graphics may be subject to special legal provisions.
The license is subject to the *Beilstein Journal of Organic Chemistry* terms and conditions: (https://www.beilstein-journals.org/bjoc/terms)

Table of contents

1. NMR and HRMS spectra S2

2. Fluorescence spectra and fluorescence quantum yield S10

3. Theoretical study S12

Orbital-weighted Fukui function S12

Calculated HOMO and LUMO energy S13

1. NMR and HRMS spectra

NMR spectra and HRMS data of compound 5a

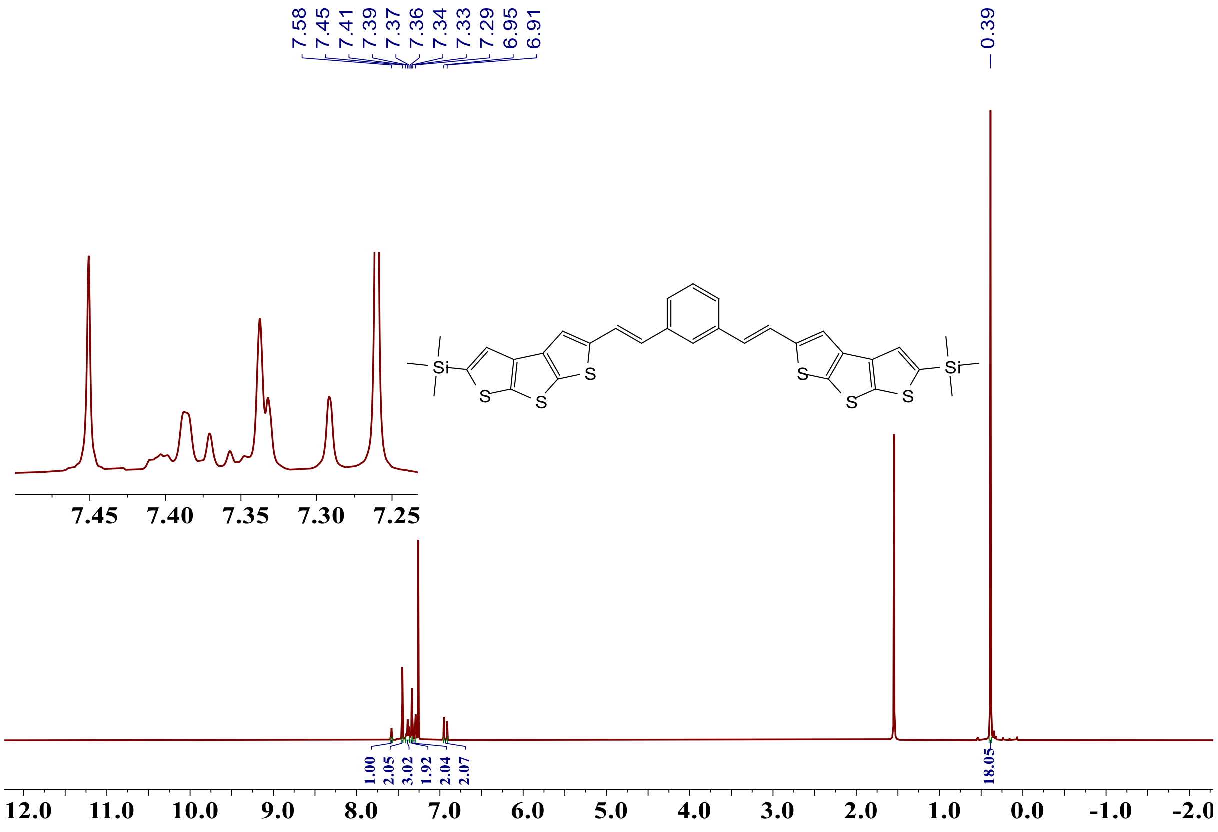

Figure S1. ^{1}H NMR (400 MHz, $CDCl_3$) spectrum of **5a**

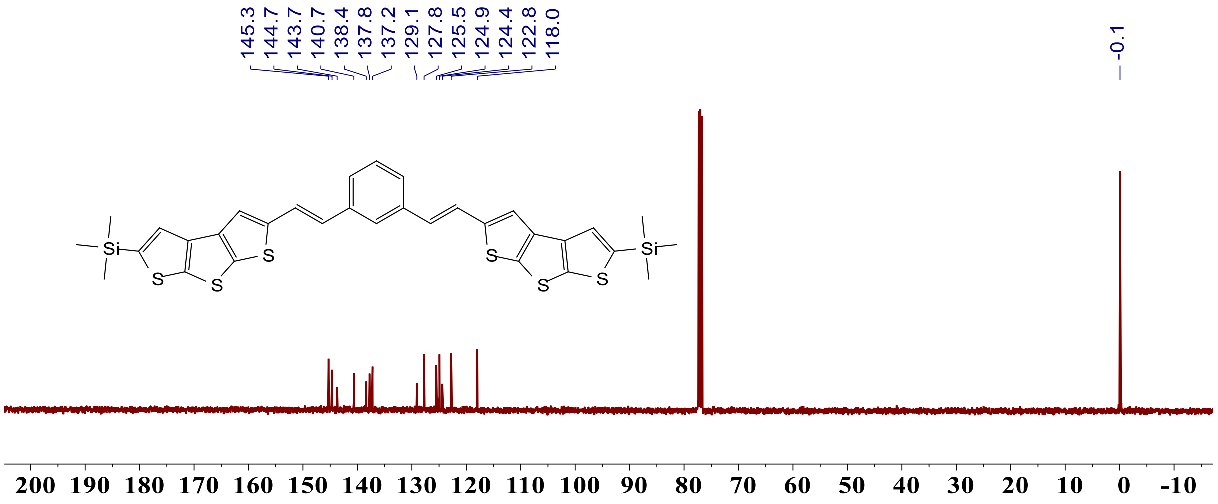

Figure S2. ^{13}C NMR (100 MHz, $CDCl_3$) spectrum of **5a**

Parameters:

Mass	Tolerance	Electron Mode	Charge	DBE Range	Max Results
662.02162 ± 0.00331	5.0 ppm	Odd/Even	+1	-0.5 - 200.0	100

Elements

C		H		N		Si		S		Na	
C	0 - 32	H	0 - 150	N	0 - 4	Si	0 - 2	S	0 - 6	Na	0 - 1

Results:

#	Formula	Mass	DBE	Abs. Error (u)	Error (u)	Error (ppm)
1	C28 H27 N4 Na Si S6	662.02214	18.0	0.00052	-0.00052	-0.78
2	C32 H30 Si2 S6	662.02048	20.0	0.00114	0.00114	1.73
3	C31 H23 N4 Na Si S5	662.01877	23.0	0.00285	0.00285	4.31
4	C30 H26 N4 Si S6	662.02455	21.0	0.00292	-0.00292	-4.42

Figure S3. HRMS data of **5a**

NMR and HRMS spectra of 5b

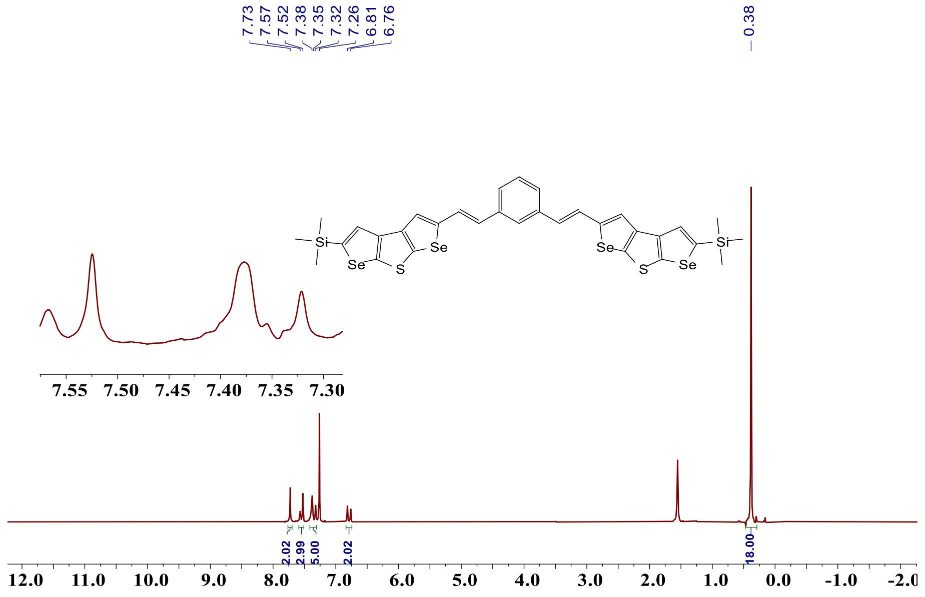

Figure S4. ^{1}H NMR (300 MHz, $CDCl_3$) spectrum of **5b**

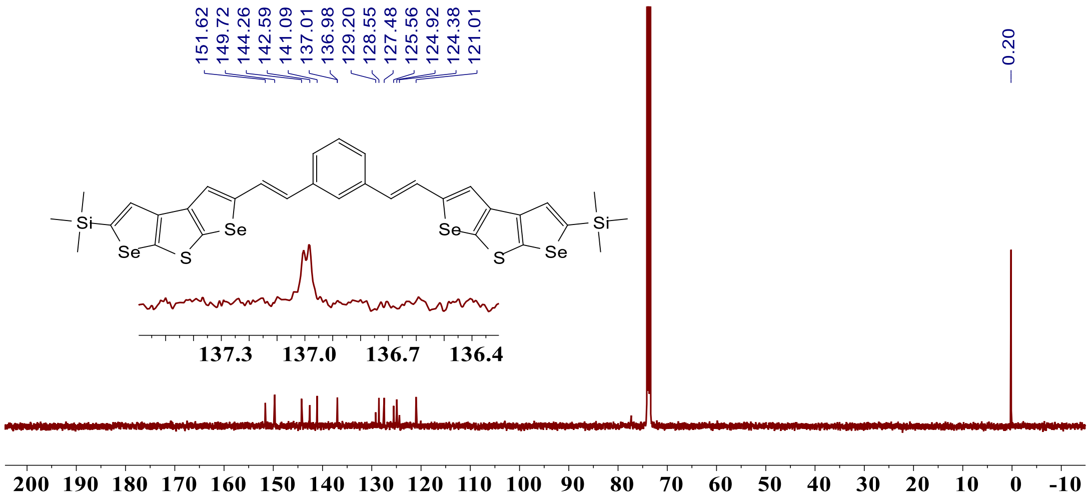

Figure S5. ^{13}C NMR (150 MHz, $C_2D_2Cl_4$) spectrum of **5b**

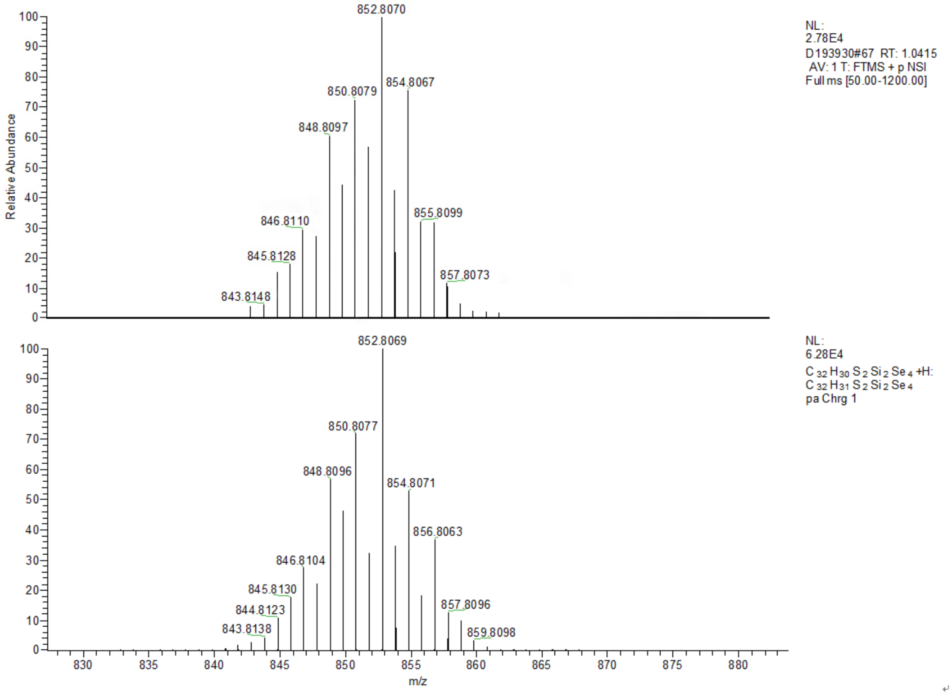

Figure S6. HRMS spectrum of **5b**

NMR and HRMS spectra of 5c

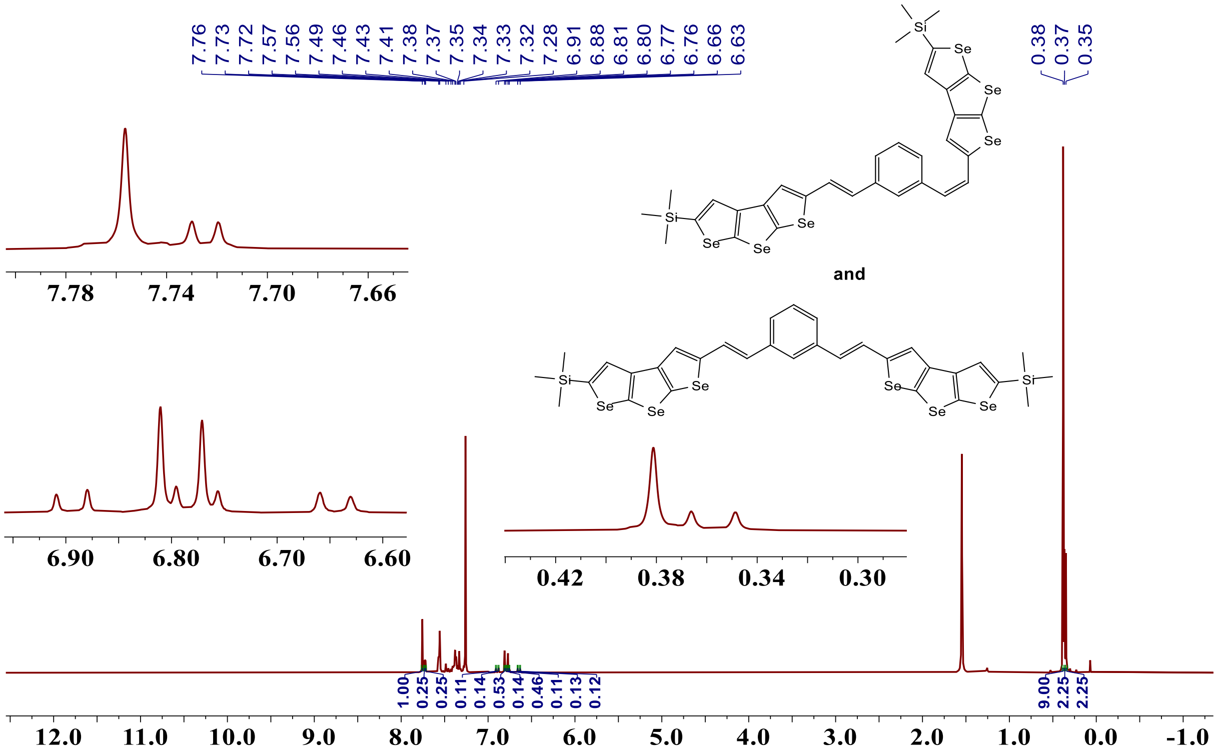

Figure S7. ^{1}H NMR (400 MHz, CDCl$_3$) spectrum of mixture of *cis*- and *trans*-**5c**

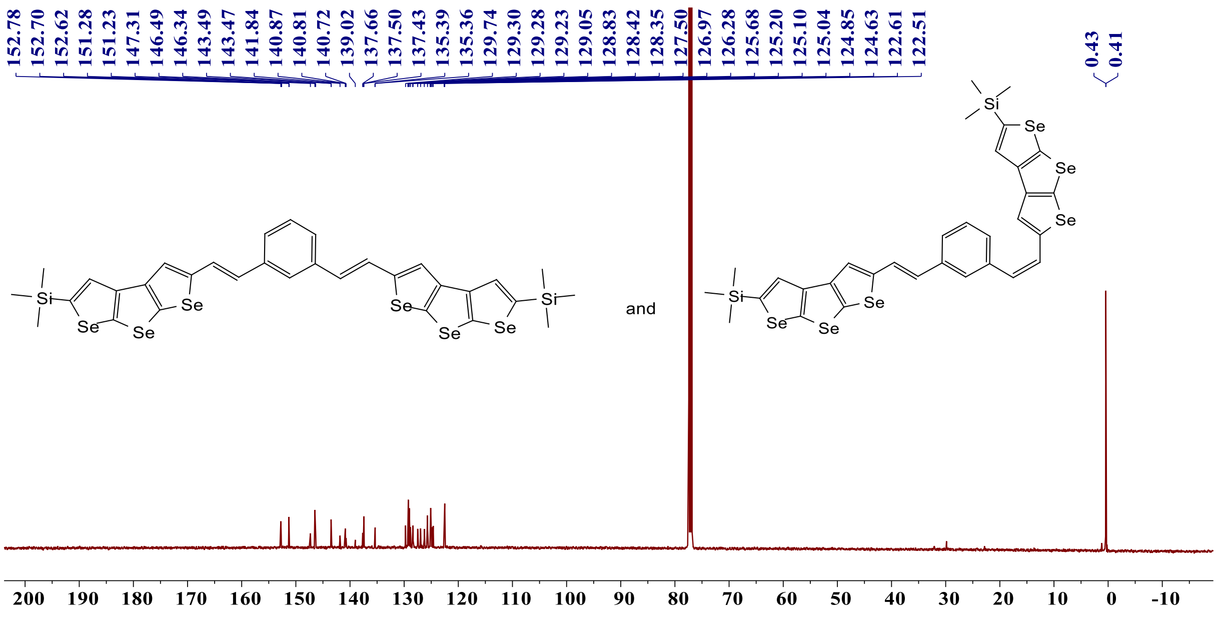

Figure S8. ^{13}C NMR (125 MHz, $CDCl_3$) spectrum of mixture of *cis*- and *trans*-**5c**

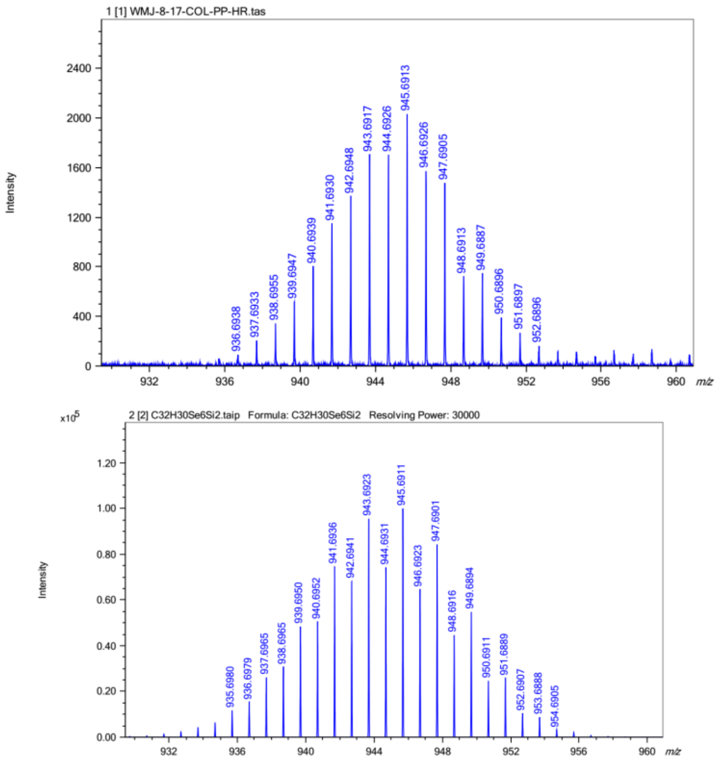

Figure S9. HRMS spectrum and data of **5c**

NMR spectra and HRMS data of DH-1

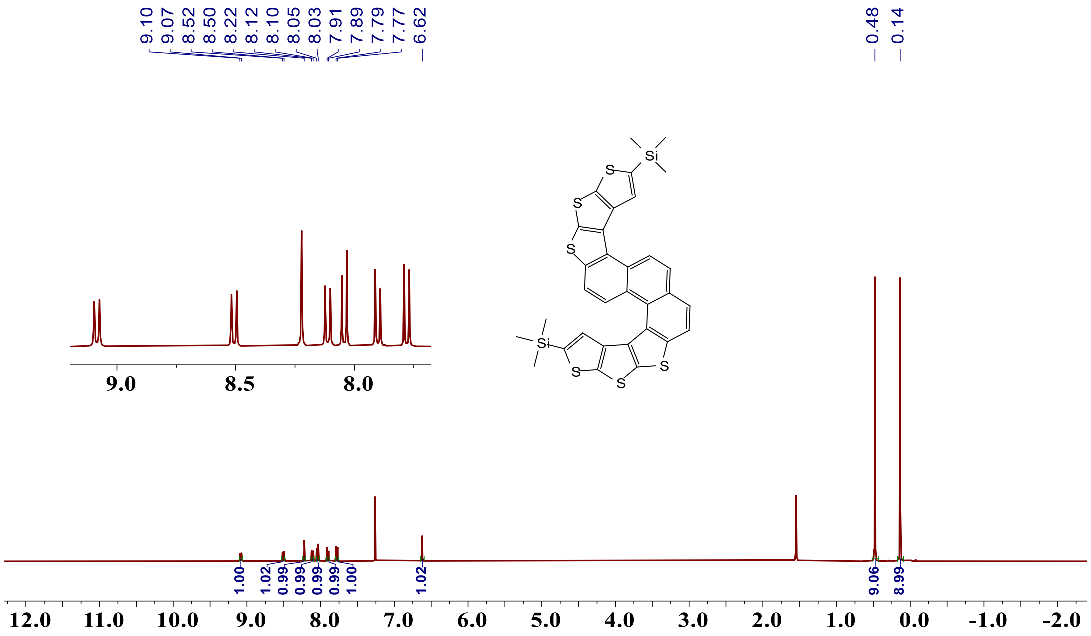

Figure S10. ^{1}H NMR (400 MHz, $CDCl_3$) spectrum of **DH-1**

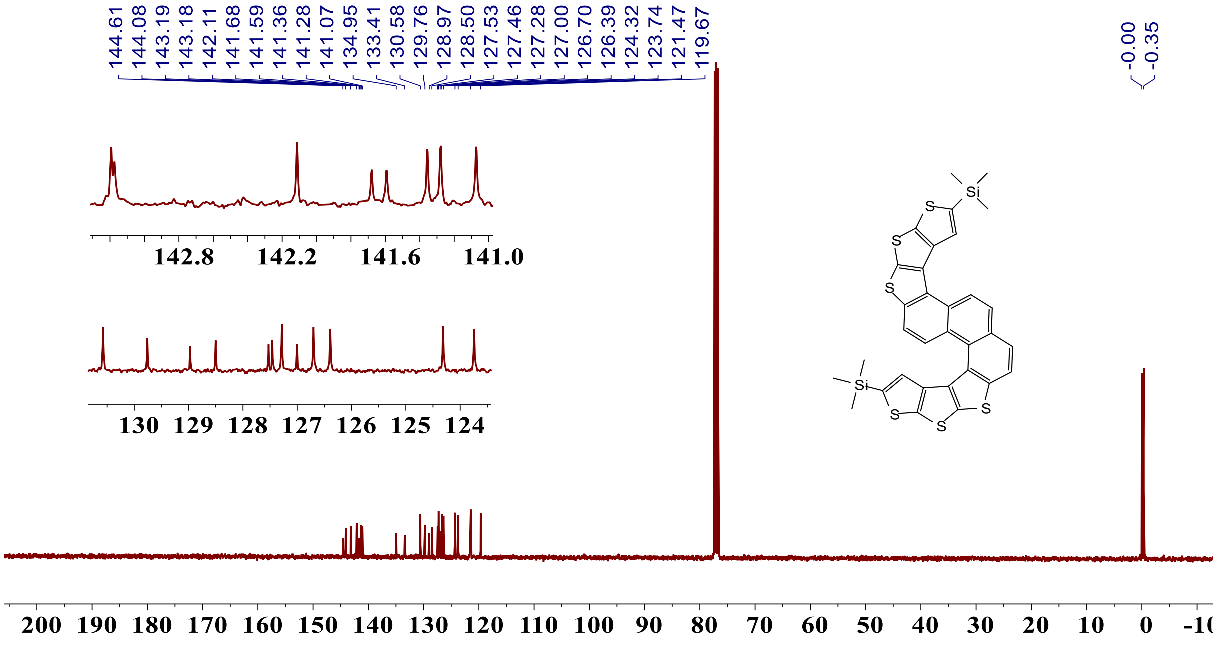

Figure S11. ^{13}C NMR (100 MHz, $CDCl_3$) spectrum of **DH-1**

Parameters:

Mass	Tolerance	Electron Mode	Charge	DBE Range	Max Results
657.98961 ± 0.00329	5.0 ppm	Odd/Even	+1	-0.5 - 200.0	100

Elements

C 0 - 32 H 0 - 150 N 0 - 4 Si 0 - 2 S 0 - 6 Na 0 - 1

Results:

#	Formula	Mass	DBE	Abs. Error (u)	Error (u)	Error (ppm)
1	C32 H26 Si2 S6	657.98918	22.0	0.00043	0.00043	0.65
2	C28 H23 N4 Na Si S6	657.99084	20.0	0.00123	-0.00123	-1.87
3	C31 H19 N4 Na Si S5	657.98747	25.0	0.00214	0.00214	3.25
4	C30 H27 Na Si2 S6	657.98677	19.0	0.00284	0.00284	4.31

Figure S12. HRMS data of **DH-1**

NMR and HRMS spectra of DH-2

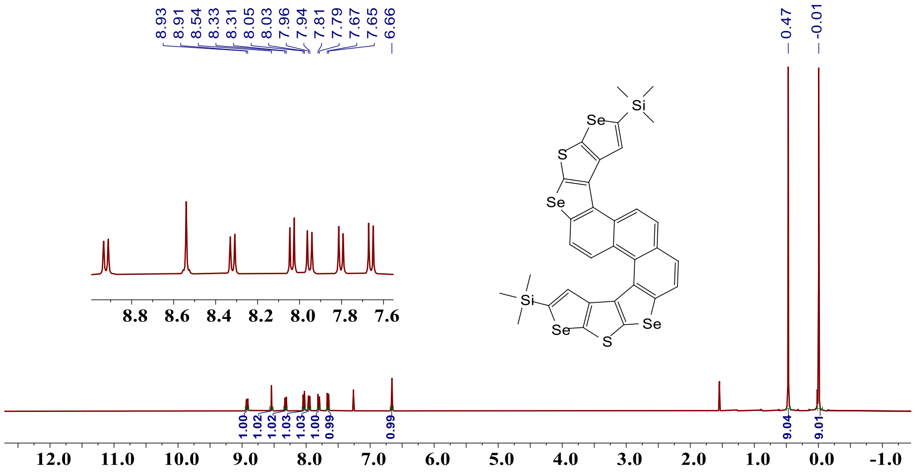

Figure S13. ^{1}H NMR (400 MHz, $CDCl_3$) spectrum of **DH-2**

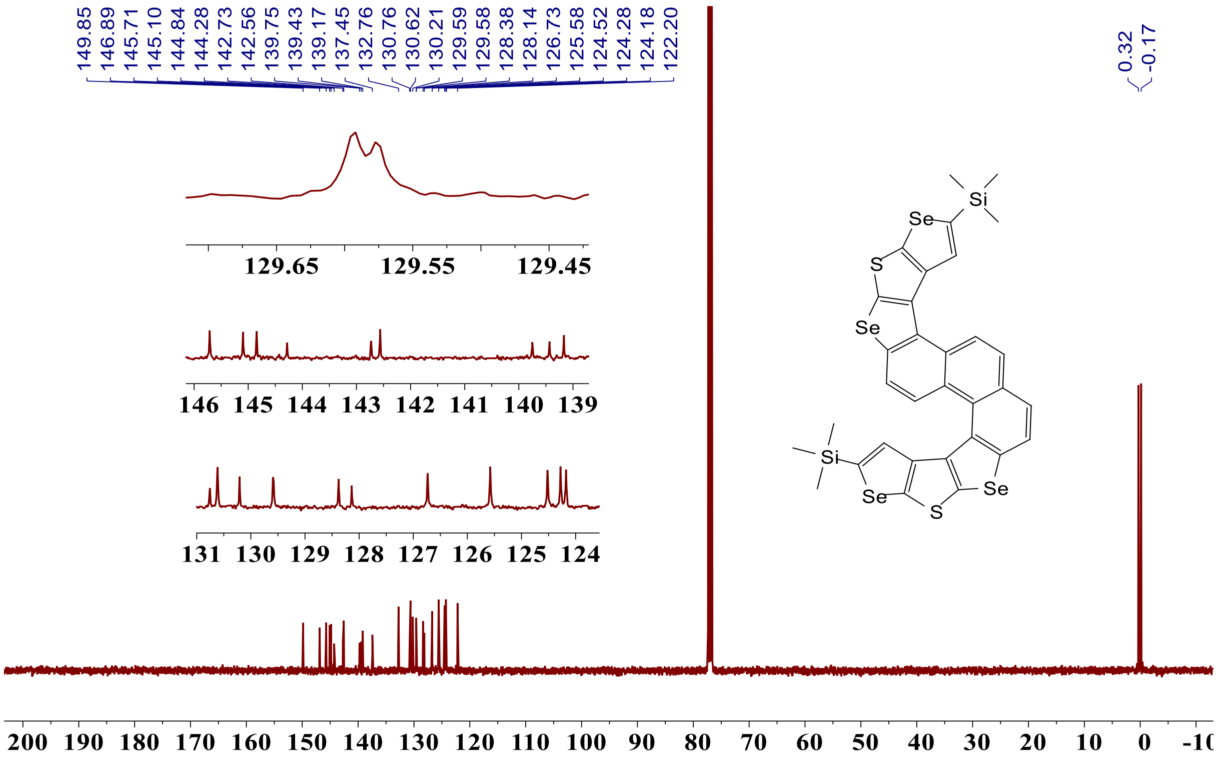

Figure S14. ^{13}C NMR (100 MHz, $CDCl_3$) spectrum of **DH-2**

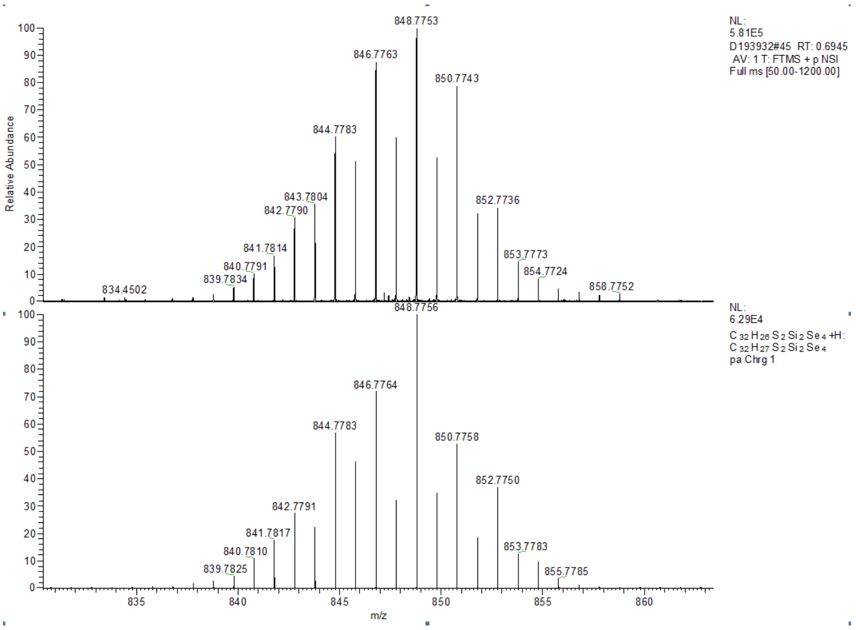

Figure S15. HRMS spectrum of **DH-2**

NMR and HRMS Spectra of DH-3

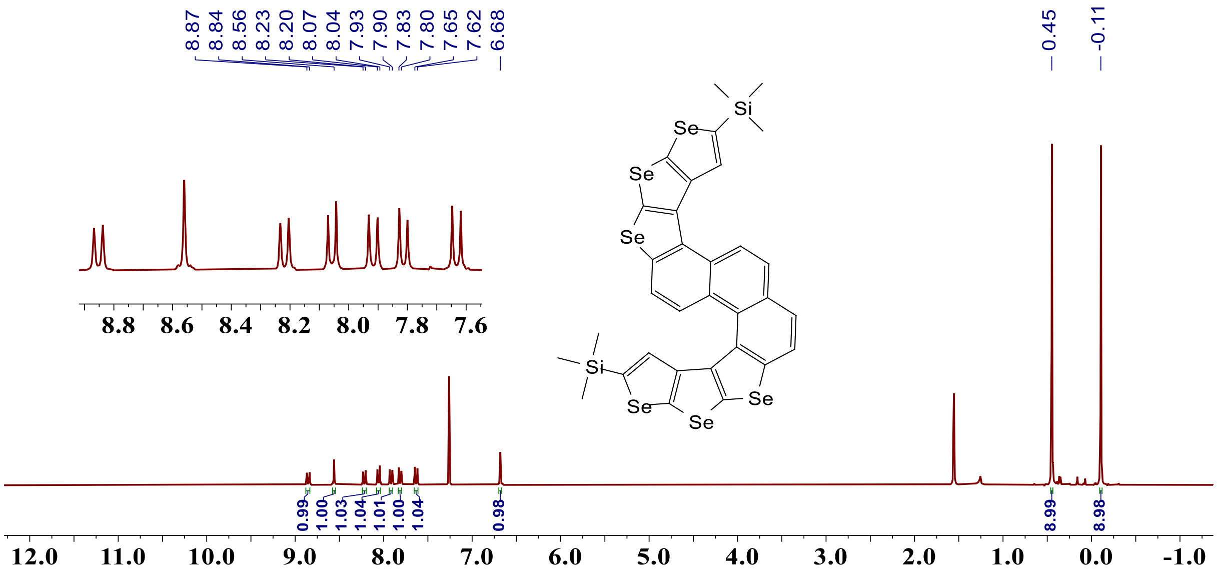

Figure S16. ^{1}H NMR (300 MHz, $CDCl_3$) spectrum of **DH-3**

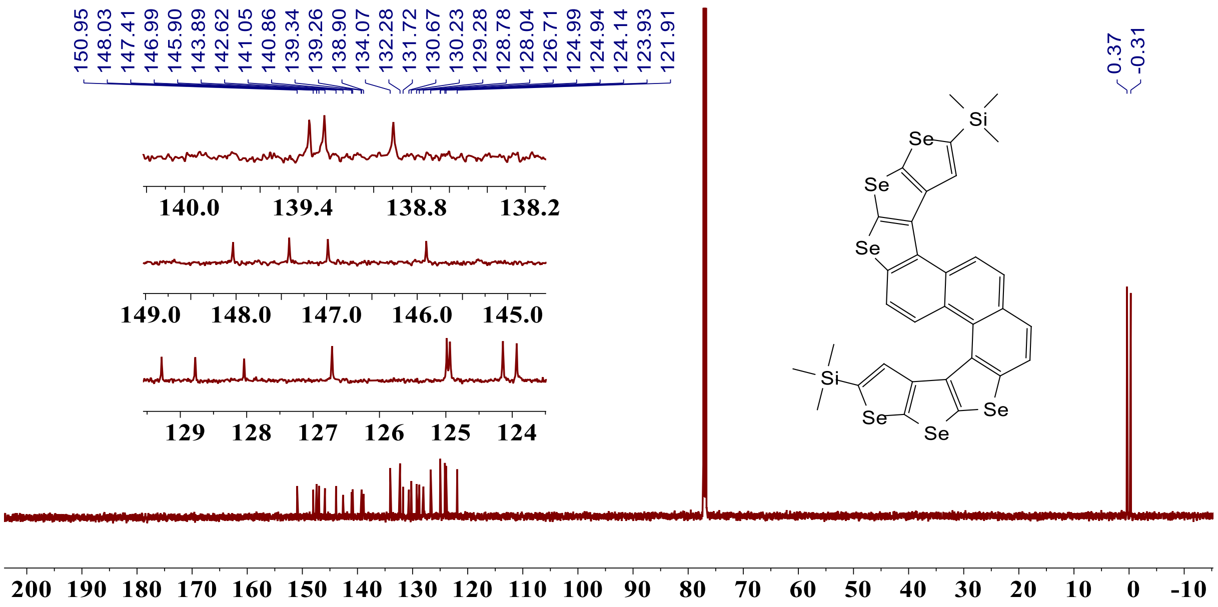

Figure S17. ^{13}C NMR (100 MHz, $CDCl_3$) spectrum of **DH-3**

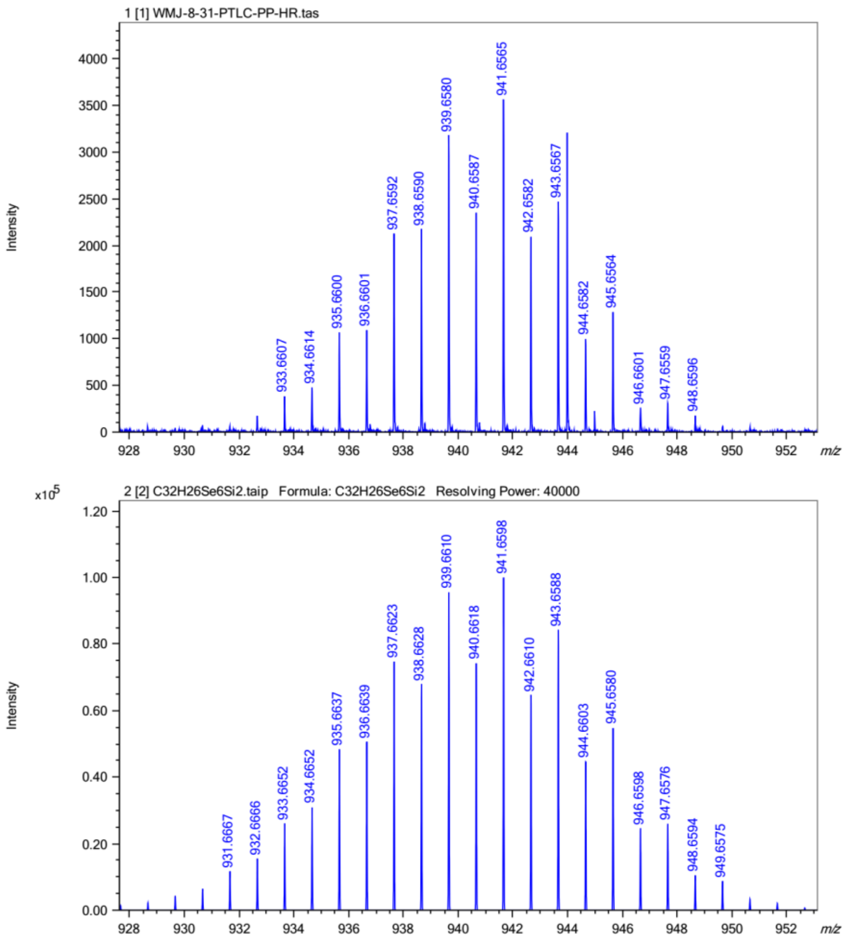

Figure S18. HRMS spectrum and data of **DH-3**

2. Fluorescence spectra and fluorescence quantum yield

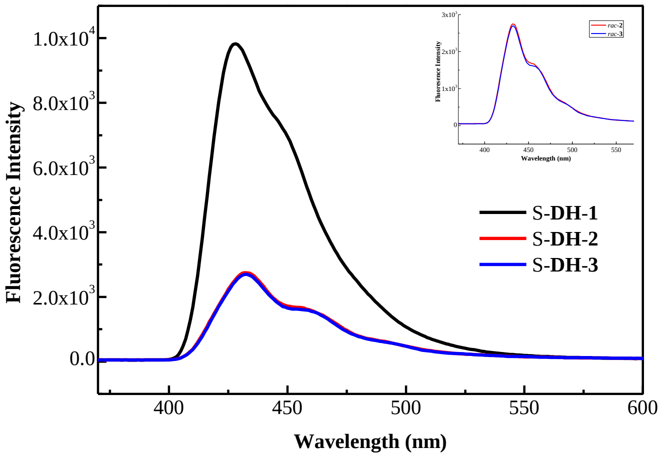

Figure S19. Fluorescence spectra of **DH-1–3** at room temperature in dichloromethane ([C] = 1×10^{-5} M, λ_{ex} = 350 nm, slit: 1/1 nm).

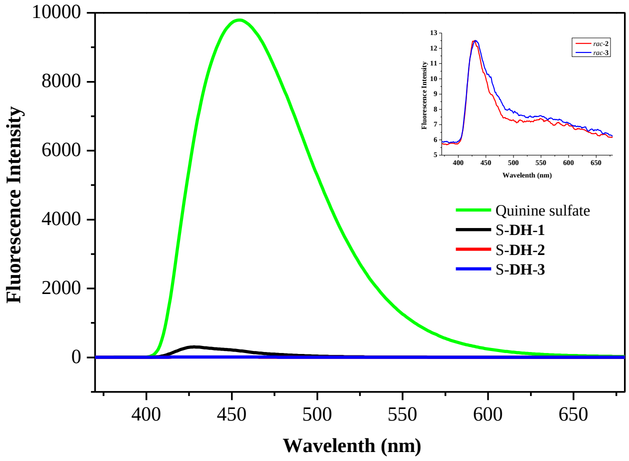

Figure S20. Excitation spectra of compound **DH-1–3** in dichloromethane and quinine sulfate dehydrate in 0.1N H_2SO_4. (λ_{ex} = 350 nm, [C] = 1 × 10^{-5} M).

Table S1. The fluorescence quantum yield (Φ) of **DH-1–3** calculated according to the formula: $\Phi_F = (n_x/n_s)^2 A_s/A_x\, D_x/D_s \Phi_s$

Compound	Peak Area(D)	Refractive index(n)	Yeild(Φ)
**DH-1**	1.6×10^4	1.42	0.0123
**DH-2**	2.3×10^3	1.42	0.0016
**DH-3**	2.4×10^3	1.42	0.0017
Quinine sulfate	8.4×10^5	1.33	0.54

3. Theoretical study

Orbital-weighted Fukui function of **5a**

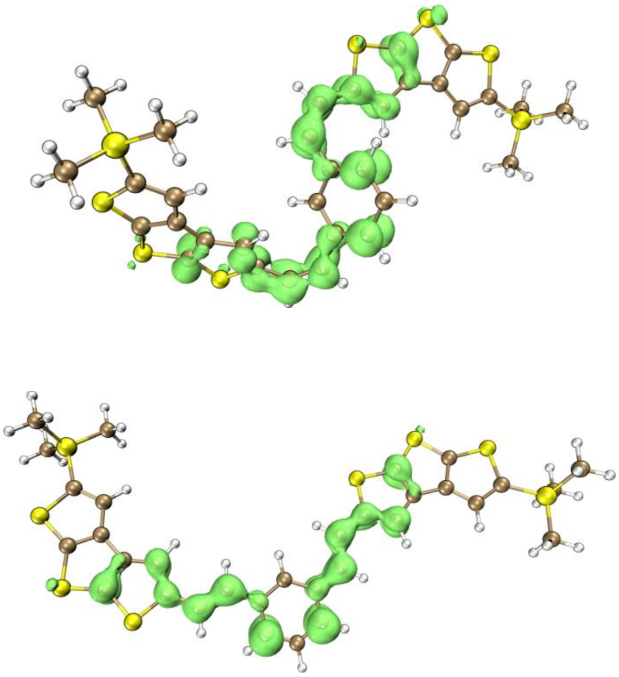

Figure S21. Orbital-weighted Fukui function of **5a**.

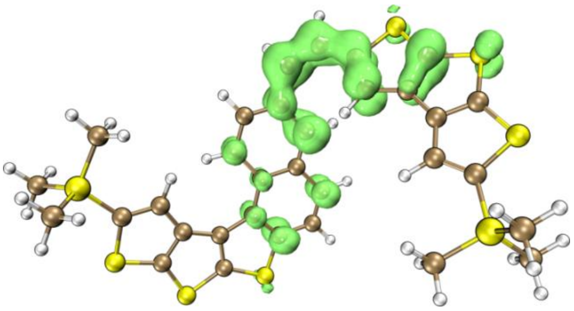

Figure S22. Orbital-weighted Fukui function of closing one benzene compound.

Calculated HOMO and LUMO energy

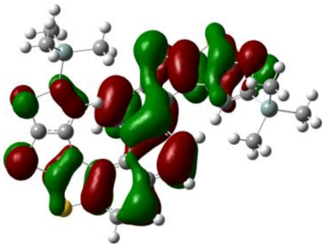

DH-1_HOMO

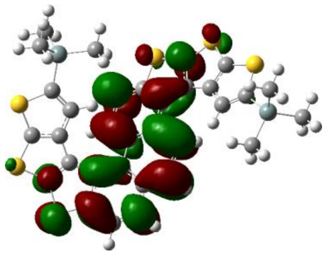

DH-1_LUMO

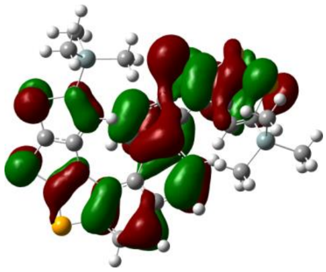

DH-2_HOMO

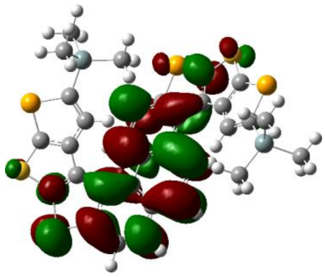

DH-2_LUMO

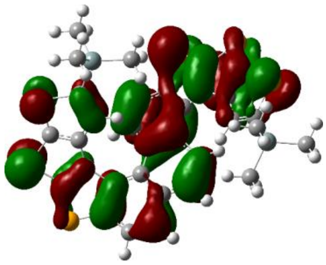

DH-3_HOMO

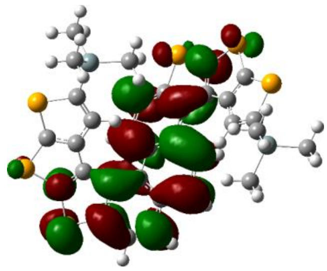

DH-3_LUMO

Figure S23. The contour plots of the HOMOs and LUMOs for **DH-1**, **DH-2**, and **DH-3**.

Table S2. Calculated HOMO and LUMO energy levels and energy gap at B3LYP/6-31G** level of theory and the optical band gaps estimated from the absorption edges.

Compounds	LUMO (eV) theory	HOMO (eV) theory	E_g (eV) theory	λ_{onset} (nm) experimental	E_g^{opt} (eV) experimental
**DH-1**	-1.42	-5.39	3.97	403	3.08
**DH-2**	-1.49	-5.32	3.83	412	3.01
**DH-3**	-1.47	-5.28	3.81	416	2.98
