# Supplementary material for: Thiophene/selenophene-based S-shaped double helicenes: regioselective synthesis and structures
Source: Beilstein J Org Chem. 2022 Jul 8;18:809–17. doi: 10.3762/bjoc.18.81 (PMC9273980; doi:10.3762/bjoc.18.81)
Supplement: File 2 — Supporting crystallographic information for compounds DH-1 and DH-2. [file Beilstein_J_Org_Chem-18-809-s002.pdf]

BEILSTEIN JOURNAL OF ORGANIC CHEMISTRY

Supporting Information

for

Thiophene/selenophene-based S-shaped double helicenes: regioselective synthesis and structures

Mengjie Wang, Lanping Dang, Wan Xu, Zhiying Ma, Liuliu Shao, Guangxia Wang, Chunli Li and Hua Wang

Beilstein J. Org. Chem. **2022,** *18,* 809–817. doi:10.3762/bjoc.18.81

Supporting crystallographic information for compounds DH-1 and DH-2

License and Terms: This is a supporting information file under the terms of the Creative Commons Attribution License (https://creativecommons.org/licenses/by/4.0). Please note that the reuse, redistribution and reproduction in particular requires that the author(s) and source are credited and that individual graphics may be subject to special legal provisions.
The license is subject to the *Beilstein Journal of Organic Chemistry* terms and conditions: (https://www.beilstein-journals.org/bjoc/terms)

Table of contents

X-ray crystallographic data for compound **DH-1** S2

X-ray crystallographic data for compound **DH-2** S5

X-ray crystallographic data

Complete crystal data for DH-1

Table S1. Crystal data and structure refinement for **DH-1**.

Identification code	**DH-1**	
Empirical formula	$C_{32}H_{26}S_6Si_2$	
Formula weight	659.07	
Temperature	150.0 K	
Wavelength	0.71073 Å	
Crystal system	Triclinic	
Space group	P-1	
Unit cell dimensions	a = 11.1290(7) Å	α = 108.362(2)°
	b = 12.1240(7) Å	β = 111.318(2)°
	c = 13.7363(9) Å	γ = 100.961(2)°
Volume	1538.32(17) $Å^3$	
Z	2	
Density (calculated)	1.423 Mg/m^3	
Absorption coefficient	0.546 mm^{-1}	
F(000)	684	
Crystal size	0.14 x 0.12 x 0.08 mm^3	
Theta range for data collection	2.284 to 25.499°	
Index ranges	$-13 \leq h \leq 13$, $-14 \leq k \leq 13$, $-16 \leq l \leq 16$	
Reflections collected	19274	
Independent reflections	5716 [R(int) = 0.1014]	
Completeness to theta = 25.242°	99.7 %	
Absorption correction	Semi-empirical from equivalents	
Max. and min. transmission	0.7457 and 0.6783	
Refinement method	Full-matrix least-squares on F^2	
Data / restraints / parameters	5716 / 0 / 367	
Goodness-of-fit on F^2	1.047	
Final R indices [I>2sigma(I)]	R1 = 0.0562, wR2 = 0.0872	
R indices (all data)	R1 = 0.1093, wR2 = 0.1101	
Extinction coefficient	n / a	
Largest diff. peak and hole	0.476 and -0.451 e.$Å^{-3}$	

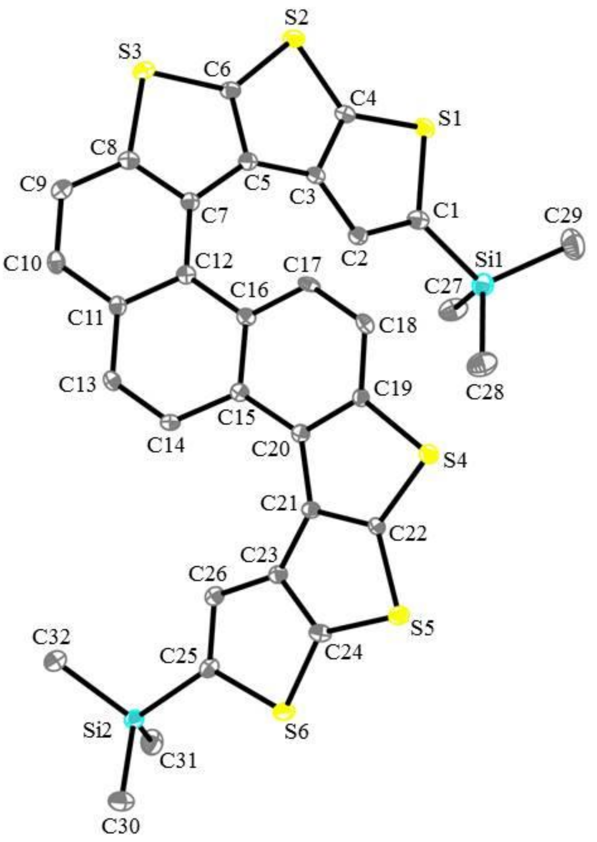

Figure S1. The crystal structures for compound **DH-1**. Carbon, sulfur, and silicon atoms are depicted with thermal ellipsoids set at 30% probability level, and all hydrogen atoms are omitted for clarity.

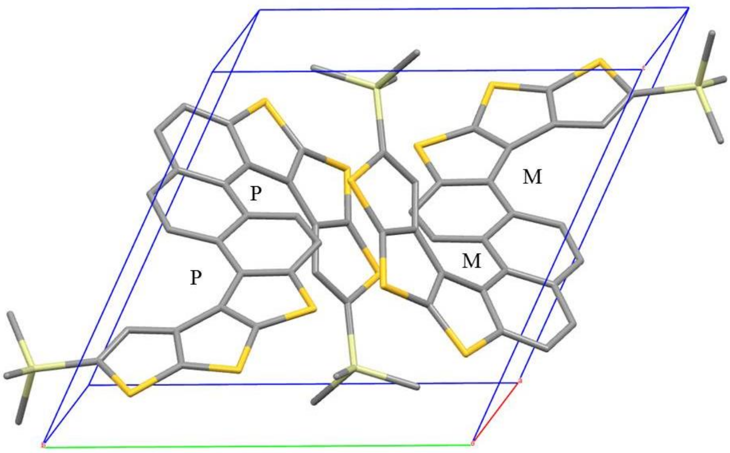

Figure S2. Molecular configuration of **DH-1** in one unit cell.

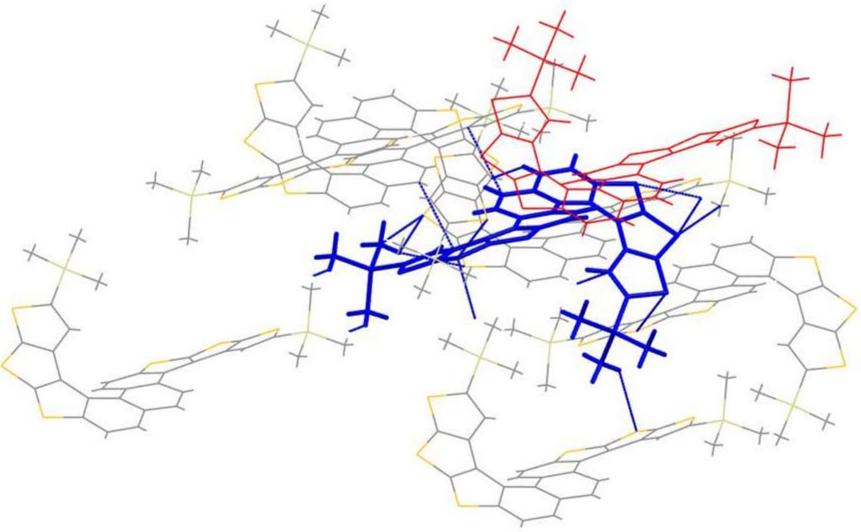

Figure S3. Multiple interactions in the crystal packings of **DH-1**.

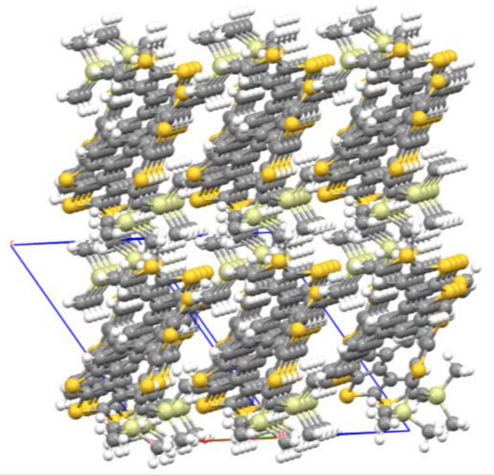

Figure S4. Molecular packing of **DH-1**.

Complete crystal data for DH-2

Table S2. Crystal data and structure refinement for **DH-2**.

Identification code	**DH-2**	
Empirical formula	$C_{32}H_{26}S_2Se_4Si_2$	
Formula weight	846.67	
Temperature	149.98 K	
Wavelength	0.71073 Å	
Crystal system	Triclinic	
Space group	P-1	
Unit cell dimensions	a = 11.0959(11) Å	α = 112.217(4)°.
	b = 12.2773(12) Å	β = 106.495(3)°.
	c = 13.5535(13) Å	γ = 99.898(3)°.
Volume	1555.7(3) $Å^3$	
Z	2	
Density (calculated)	1.807 Mg/m^3	
Absorption coefficient	4.951 mm^{-1}	
F(000)	828	
Crystal size	0.21 x 0.17 x 0.12 mm^3	
Theta range for data collection	2.288 to 28.326°.	
Index ranges	$-14 \le h \le 14$, $-15 \le k \le 16$, $-18 \le l \le 18$	
Reflections collected	23440	
Independent reflections	7708 [R(int) = 0.0726]	
Completeness to theta = 25.242°	99.7 %	
Absorption correction	Semi-empirical from equivalents	
Max. and min. transmission	0.7457 and 0.5103	
Refinement method	Full-matrix least-squares on F^2	
Data / restraints / parameters	7708 / 0 / 367	
Goodness-of-fit on F^2	1.027	
Final R indices [I>2sigma(I)]	R1 = 0.0443, wR2 = 0.0999	
R indices (all data)	R1 = 0.0690, wR2 = 0.1133	
Extinction coefficient	n/a	
Largest diff. peak and hole	1.175 and -0.880 e.$Å^{-3}$	

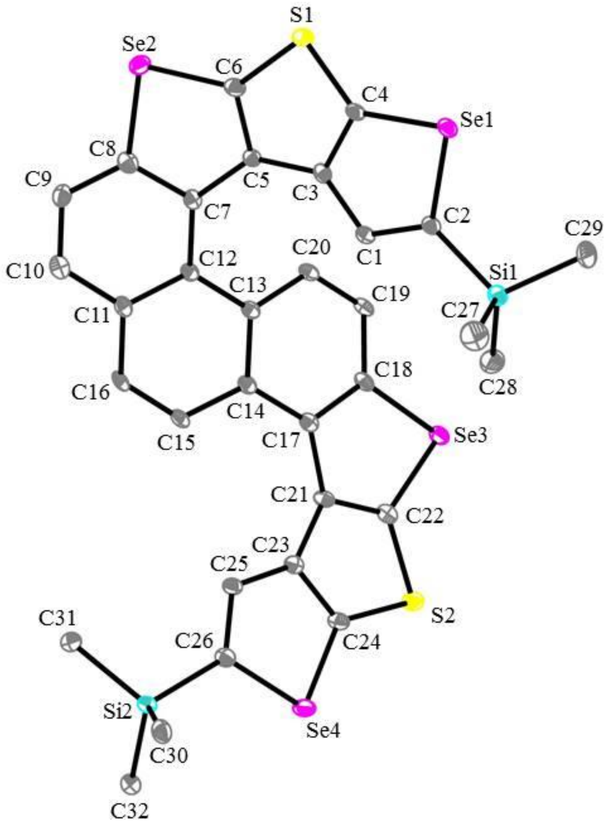

Figure S5. The crystal structures for compound **DH-2**. Carbon, selenium, sulfur, and silicon atoms are depicted with thermal ellipsoids set at 30% probability level, and all hydrogen atoms are omitted for clarity.

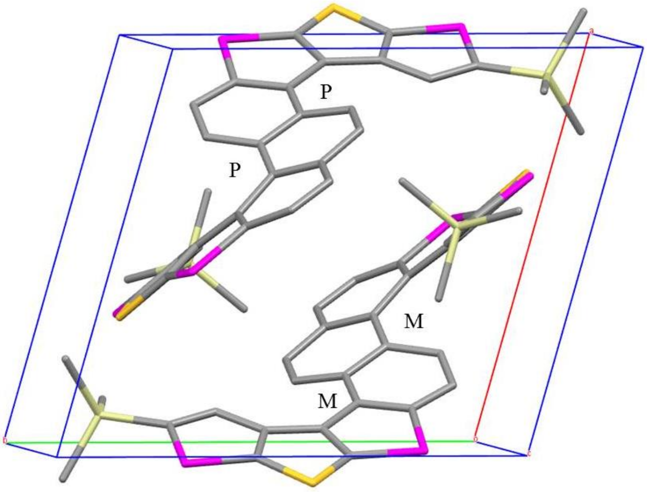

Figure S6. Molecular configuration of **DH-2** in one unit cell.

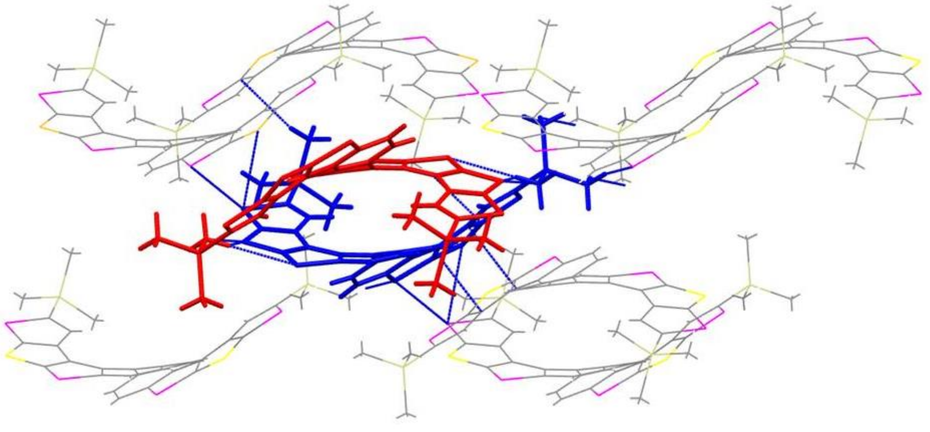

Figure S7. Multiple interactions in the crystal packings of **DH-2**

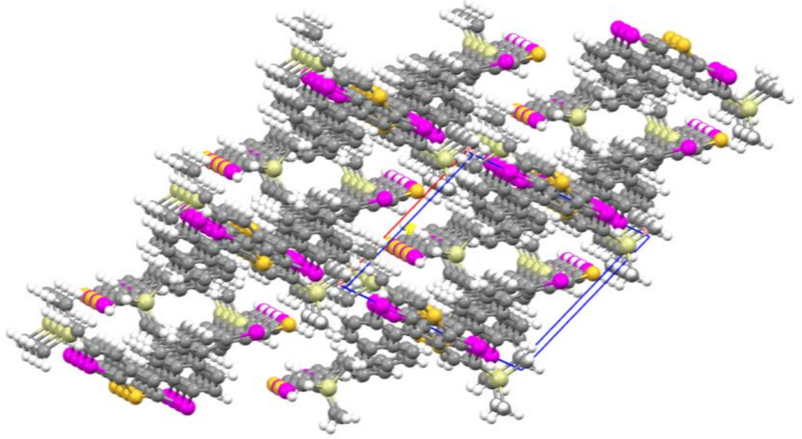

Figure S8. Molecular packing of **DH-2**.
